# Aortic Stenosis as a Mechanical Stressor and Tissue Energetics: Consistent Clue with Hypertensive Stress Septal Sign

**DOI:** 10.3390/jcm15020623

**Published:** 2026-01-13

**Authors:** Fatih Yalcin, Nagehan Kücükler, Boran Cagatay, M. Roselle Abraham, Theodore P. Abraham, Mario J. Garcia

**Affiliations:** 1 Department of Cardiology, UCSF HEALTH, School of Medicine, Cardiac Imaging, San Francisco, CA 94158, USA; 2 Department of Cardiology, Johns Hopkins Medical Institutions, School of Medicine, Cardiac Imaging, Baltimore, MD 94158, USA; 3 Department of Cardiology, Montefiore Medical Center, Albert Einstein College of Medicine, Bronx, NY 10461, USA

**Keywords:** aortic stenosis, Stress Septal Sign, left ventricular remodeling, basal septal hypertrophy, metabolism

## Abstract

**Background:** Hemodynamic overload induces left ventricular remodeling and heart failure across various clinical presentations. While geometric remodeling is classically associated with increased vascular resistance in hypertension, distinct patterns emerge under the mechanical stress of aortic stenosis (AS). **Concept:** The “Stress Septal Sign” (Triple S) represents a marker of stress-mediated hemodynamic overload driven by diverse stimuli, ranging from mechanical stress in AS to emotional triggers in acute stress cardiomyopathy. Within this spectrum, Stressed Heart Morphology describes a specific phenotype characterized by a predominant and hyperdynamic LV septal base. **Results:** Chronic hemodynamic stress in severe AS results in prominent basal septal hypertrophy. This remodeling is characterized by distinct tissue energetics: hypermetabolic activity at the basal septum contrasted with reduced metabolic activity or hypokinesis in the apical regions. These findings on myocardial geometry, function, and energetics align with the adaptive phase of LV remodeling. **Conclusions:** The presence of adaptive myocardial basal tissue suggests an advanced remodeling stage that may require timely therapeutic intervention in severe AS. Therefore, identifying these specific tissue characteristics offers a unifying imaging paradigm (Triple S) for assessing cardiac stress, independent of the primary etiology.

## 1. Introduction

Aortic stenosis (AS) constitutes a pervasive and progressive public health challenge, characterized by an earlier onset in men relative to women and a steadily increasing annual incidence. This results in an estimated prevalence of approximately 11–18% within the United States population, underscoring its substantial impact on aging demographics [1,2]. Concurrently, hypertensive heart disease has established itself as a major global health concern, with rising mortality rates and a prevalence of approximately 18.6 million cases worldwide. In the United States, systemic hypertension affects nearly one-third of the adult population, contributing to a multifaceted array of cardiovascular risks that exacerbate the overall burden of associated pathologies [3,4]. These epidemiological patterns emphasize the imperative for heightened awareness, proactive screening initiatives, and comprehensive management strategies to alleviate the societal and economic ramifications of these interrelated conditions. Beyond their overlapping epidemiological impact, these conditions converge on a shared hemodynamic pathway that fundamentally alters myocardial structure.

At the pathophysiological core of these co-prevalent conditions lies chronic pressure overload, which serves as the primary mechanism driving left ventricular (LV) remodeling in both hypertension and AS [5,6]. AS represents the principal mechanical cause of increased afterload, triggering compensatory LV remodeling to normalize wall stress [6]. Clinically, severe AS is associated with significant morbidity, including angina, syncope and congestive heart failure, and mortality. Historically, symptomatic severe AS carried a poor prognosis with fixed survival durations. However, in the modern era aortic valve replacement remains the definitive treatment to modify disease progression and improve survival. Functional analysis of myocardial tissue characteristics provides valuable data for monitoring AS patients [6]. Consequently, identifying universal imaging biomarkers that correlate with disease phases could facilitate timely and effective surgical or interventional decision-making. Thus, determining the precise extent of myocardial involvement in AS is clinically vital [7].

**Figure 1:**
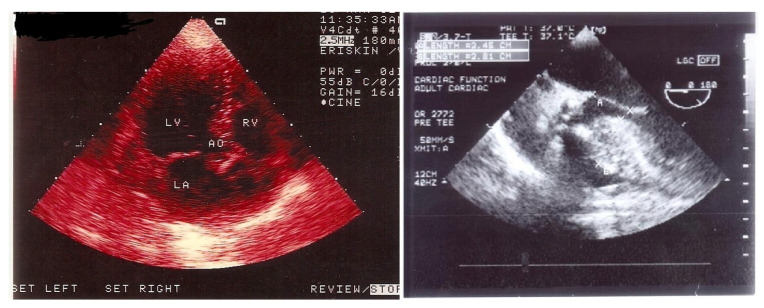
Thickened septal base on the transthoracic echocardiography in a patient with AS (LV: Left ventricle, RV: Right ventricle, LA: Left atrium, AO: Aortic valve outflow tract) (**left**) and the severely thickened septal base by the transesophageal echocardiography in another patient with severely stenotic AS (**right**).

Quantitative functional tissue evaluation, which has proven beneficial in various heart diseases [8] and is strongly recommended for AS. Previous research suggests that tissue function in mild to moderate AS remains comparable to healthy subjects [9] but deteriorates as LV mass increases and stenosis severity worsens. Notably, symptomatic aortic valve stenosis is frequently accompanied by basal septal hypertrophy (BSH), a distinct remodeling phenotype reported in approximately 10% of patients, reflecting early regional adaptation to pressure overload [10,11]. Echocardiographic imaging has gained importance in defining remodeling stages, ranging from localized basal septal hypertrophy to global LV hypertrophy (LVH) and dysfunction [12]. In addition to stenotic valve assessment [13], echocardiography with both transthoracic and transesophageal options is widespread used to assess the severity of remodeling in AS (Figure 1). In fact, the potential relation between the severity of LV basal septal involvement and the duration of the disease course has been pointed out in patients with hypertensive heart disease [14].

Despite these advances, the specific impact of flow dynamics on basal septal geometry in AS remains underappreciated. Therefore, this study aims to elucidate the relationship between Stressed Heart Morphology (SHM) and adaptive remodeling under chronic pressure overload. We specifically sought to demonstrate how turbulent LV outflow tract flow contributes to basal septal changes, utilizing the “Stress Septal Sign” (Triple S) as a unifying imaging marker. This approach offers a novel perspective on characterizing the transition from adaptive remodeling to heart failure in severe AS.

## 2. Clinical Conditions and Stressed Heart Morphology

The assessment of segmental LV remodeling through real-time three-dimensional echocardiography has demonstrated that patients subjected to pressure overload develop a distinct and non-uniform morphological heterogeneity. To precisely visualize and quantify these segmental nuances, we previously established a volumetric index that partitions the LV cavity along its longitudinal axis into three equal components comprising the base, mid-cavity, and apex. In earlier investigations conducted two decades ago, this detailed segmentation revealed distinct geometric phenotypes; specifically, secondary LVH resulting from AS and hypertension was consistently associated with a diminished basal intracavity volume resembling an ampulla shape. This contrasts significantly with the morphology observed in hypertrophic cardiomyopathy, which is characterized by a reduction in mid-segmental volume that reflects a catenoid geometry [15].

SHM was originally described based on the recognition that a predominantly developed septal base constitutes a geometric similarity and morphologic conjunction across a spectrum of clinical conditions, ranging from emotion-mediated acute stress cardiomyopathy to chronic increased afterload states. Building on this framework, we recently highlighted the presence of a distinct mechanical component of SHM in patients with AS that complements the previously established emotional and functional components [16]. Furthermore, comparative imaging analysis reveals a distinct divergence in remodeling patterns; contrary to the regular LV remodeling distribution seen in animal studies via third-generation microscopic ultrasonography, human data presents with an extremely heterogeneous morphology [17].

**Figure 2:**
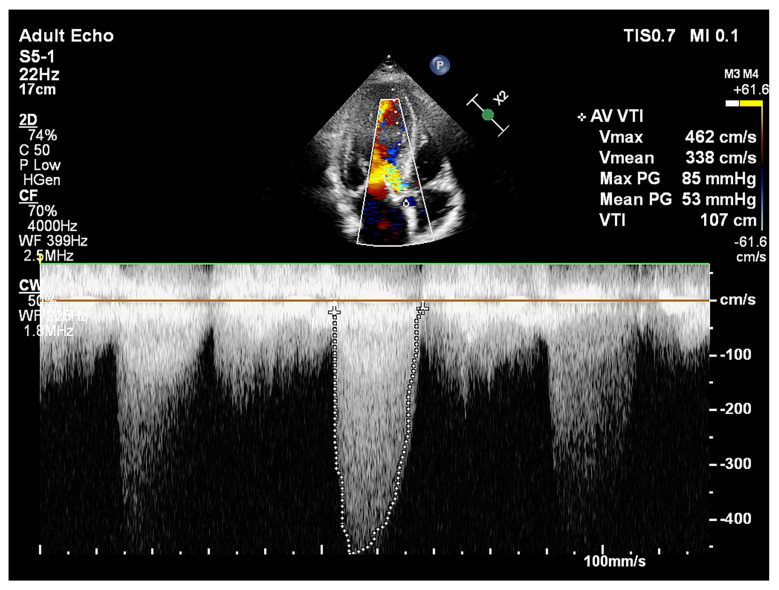
Echocardiographic assessment of a symptomatic patient with AS and SHM. The image demonstrates increased transvalvular aortic gradients and turbulent blood flow in the left ventricular outflow tract. That the turbulent flow directly contacts the predominant septal base, which may contribute to SHM development under chronic AS-mediated stress.

In the context of AS, high-pressure gradients generate turbulent flow that directly contacts the LV base (Figure 2). This mechanical force likely serves as a potent stimulus driving the progression of SHM.

**Figure 3:**
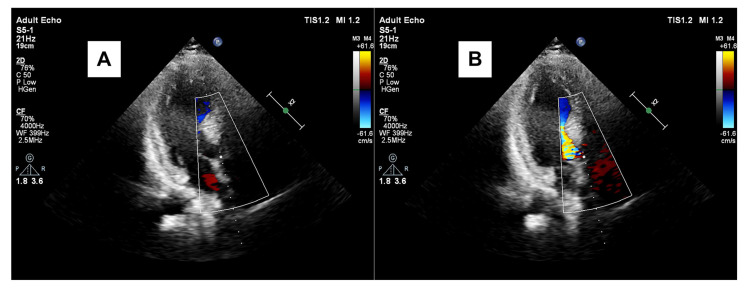
Thickened septal base at diastole in a hypertensive patient with SHM (**A**) and systolic turbulent blood flow and prominence of septal base in the same patient (**B**).

We previously demonstrated that focal hypertrophy of LV base or BSH (Figure 3) is related to high-pressure heart rate product under stress induction in hypertension. We discussed the importance of exaggerated hypertension under stress [18,19]. Tissue Doppler imaging in these patients confirms that hyperdynamic myocardial function accompanies the increased vascular resistance characteristic of stress [20]. Besides the predominant BSH, dynamic LV outflow obstruction could be determined in AS [21], which could be the case in patients with hypertensive heart disease [18,19,20]. A hypercontractile base is also a hallmark of acute stress cardiomyopathy, contrasting with the characteristic mid-apical ballooning and hypokinesis [17,22]. Thus, SHM manifests independent of the specific stressor type [21,22,23,24,25], and the tissue aspects of this specific segmental remodeling are becoming clinically more important and possibly related to tissue adaptation that compensates for increased hemodynamic stress [16,26,27,28].

## 3. Myocardial Dysfunction and Transition to Heart Failure

Hypertension is the major risk factor for cardiovascular and cerebrovascular diseases and a principal driver of LV remodeling. Chronic pressure overload induces regional myocardial structural changes that ultimately progress to global LV dysfunction [17,29]. Using longitudinal imaging, we previously demonstrated that BSH represents an early and reproducible imaging marker of this remodeling process under both physiologic and pathologic stress, preceding the development of LVH and heart failure [30].

Advanced cardiac imaging techniques have substantially improved the quantitative characterization of hypertensive remodeling. Real-time three-dimensional echocardiography and stress-based imaging modalities provide detailed assessment of LV geometry, regional mechanics, and myocardial performance [15,31,32]. In the early stages of hypertensive heart disease, LV hypercontractility is a consistent finding [32,33], which we confirmed using combined tissue Doppler imaging and dobutamine stress echocardiography [20].

Importantly, effective antihypertensive therapy can slow LVH progression and reduce myocardial mass, supporting the concept that early hypertensive remodeling is, at least in part, reversible [34,35]. However, echocardiographic and nuclear studies document that LV hypocontractility develops with progression of hypertensive heart disease and leads to an increased risk for cardiovascular events [36,37].

In hypertensive LVH patients with preserved or supranormal LV function, stress-corrected midwall or endocardial wall fractional shortening can be impaired. Recently, it has been demonstrated that improvement in midwall or endocardial wall fractional shortening by aggressive antihypertensive treatment is associated with reduced cardiovascular events and heart failure incidence [35]. Effective treatment possibly keeps global myocardial function preserved, which is also confirmed by other imaging techniques using tissue Doppler imaging or mitral annular motion by real-time 3-dimensional echocardiography, which reflects global LV function [38]. Preservation of global LV function during the compensated stage is also supported by experimental data demonstrating maintained cardiac energy metabolism at the LVH stage prior to overt heart failure in Dahl salt-sensitive rats [39].

Heart failure with preserved ejection fraction (HFpEF) is a relatively new clinical entity, and almost half of the total heart failure patients. Because most of the HFpEF patients have systemic hypertension, HFpEF is usually accepted as a hypertension-mediated phenomenon. Preserved global systolic function has been documented by freehand 3-dimensional echocardiography at rest in patients with HFpEF [40]. Despite normal global EF, it is emphasized that cardiac contractile abnormalities and severe diastolic dysfunction are more common in HFpEF than in patients with hypertensive LVH [41]. LVH is the main target for novel antihypertensive therapy, and the severity of LVH may help clarify pathophysiology and define a target HFpEF population for future trials [42]. Unfortunately, elderly patients with HFpEF who are on rational medical management have a low prevalence of LVH, which appears to be important for elderly patients as reported by I-Preserve [43].

Fibrotic tissue accumulation progressively decreases diastolic filling and results in impaired LV contractility in HFpEF [44]. Because both conditions have preserved global EF at rest, it is crucial to separate hypertensive LVH from HFpEF. In hypertensive patients, evaluation of contractile reserve may be beneficial for monitoring for any progression to HF. Therefore, using the diagnostic techniques optimally is remarkably important in early diagnosis and early as well as long-term effective treatment in patients with hypertensive heart disease. Very recently, it has been presented that myocardial fibrosis detected by magnetic resonance imaging is associated with diastolic heart failure in hypertensive heart disease [45].

Age appears to critically modify this trajectory. We have shown that contractile reserve during stress is preserved in younger hypertensive patients with LVH, whereas stress-induced contractility becomes markedly blunted in patients over 70 years of age [16,45]. Comprehensive imaging studies document that LV contractility parameters are blunted by stress induction in the HFpEF group as well [46,47].

It has been reported that AT1 receptor blockage used in this subset of patients may reduce the progressive fibrosis [38]. Specific therapy focusing on fibrosis in addition to blood pressure control in HFpEF patients will require longer follow-up to see the beneficial effects. However, these elderly patients may often die of other non-cardiac causes before benefiting from these drugs with anti-proliferative and anti-fibrotic actions.

Hypertensive heart disease is a progressive process that includes different stages. The early stage of the disease course is usually accepted as a reversible process and can be regressed effectively by antihypertensive medical therapy. We and the others have pointed out that hypertensive heart before HF development is associated with hypercontractility and preserved cardiac function [20,38,39]. Although very clear documentation of blunted cardiac function in HFpEF [46,47], there is a debate on cardiac contractile ability in the hypertension stage before the development of HF. Because some signs have been pointed out in elderly hypertensives regarding cardiac contractile abnormalities, research on the consequences of this very prevalent problem in elderly hypertensives will be important [48].

The missing link that causes the transition from hypertensive heart disease to HFpEF has not been clearly elucidated yet. Preserved LV contractility [38] in hypertensive LVH may be a consistent finding with the preserved cardiac energy metabolism at the LVH stage before heart failure development as shown in Dahl salt-sensitive rats [39]. In fact, it has been shown that myocardial metabolic changes determined by MRI are related to functional impairments in hypertensives, as indicated by a reduced LVEF [49]. Despite the presence of fibrosis determined by MRI in the aging heart compared to the young one, there is no study that compares the presence of fibrosis in aged hypertensive heart vs. young hypertensive heart [50]. Furthermore, this potential difference can clearly explain the mechanism of blunted contractility under stress in the elderly, in contrast to the young heart with hypertension [20,48]. MRI has been used to document the presence of fibrosis in hypertensive hearts [51,52]. In hypertensive LVH, PET shows a good adaptation to the increased baseline workload with maintained maximal cardiac performance that is the consistent finding with preserved contractility before HF development [53]. Moreover, this research on the tissue details of the progressive hypertensive process by the state-of-the-art medical facilities, including PET and MRI, may also contribute to the interpretation of therapeutic failure in elderly patients with hypertensive heart disease and may prepare a base for further research in this field.

## 4. Animal Validation Studies in SHM

Since all these previous clinical observations relating to SHM are cross-sectional, we conducted longitudinal stress induction studies on the animal model using 3rd generation microscopic ultrasonography [30]. Microimaging [28] showed the focally thickened septal base as the first remodeled LV segment as the validation of the early imaging biomarker in both physiologic and pathologic stress due to animal exercise training and transverse aortic constriction (TAC), respectively [30]. Nevertheless, TAC as the clinic model of AS, results in the hyperdynamic fluid dynamics with increased intracavitary gradients in animals [54]. Animal validation data confirmed the adaptive phase findings of LV remodeling under stress before tissue maladaptation [16,26,27,28].

## 5. Acute Stress Cardiomyopathy and SHM

Acute stress cardiomyopathy represents the most rapidly developing entity within the spectrum of stress-mediated heart disease [22,55,56]. Although traditionally regarded as an acute and self-limiting disorder, accumulating evidence supported by advanced imaging techniques indicates that Takotsubo syndrome more accurately reflects the acute manifestation of a chronically evolving myocardial vulnerability [57,58]. While the clinical presentation is abrupt and frequently mimics acute coronary syndrome, the underlying myocardial substrate appears to develop progressively through subtle structural, functional, and hemodynamic alterations [59]. These changes, described within the framework of the Triple S paradigm, include a small left ventricular cavity, mild basal septal hypertrophy, dynamic intraventricular gradients, altered ventricular compliance, and chronic pressure loading associated with hypertension or age-related remodeling. Such features often remain clinically silent until an acute emotional, mechanical, or metabolic stressor acts as the final trigger, unmasking the vulnerable myocardial phenotype and precipitating the characteristic pattern of apical ballooning.

Pathophysiologically, acute stress cardiomyopathy shares key mechanisms with hypertensive heart disease, including coronary microvascular dysfunction and catecholamine-mediated cardiotoxicity [22,55,56]. While chronic hemodynamic stress in hypertensive heart disease and aortic stenosis is associated with relatively greater mid-apical myocardial involvement secondary to left ventricular hypertrophy, acute stress cardiomyopathy is characterized by more pronounced mid-apical dilation in the presence of normal coronary arteries [60,61]. We hypothesize that the tissue characteristics of SHM are driven by the non-uniform distribution of sympathetic receptors; specifically, the dense sympathetic innervation at the LV base likely predisposes this segment to hypercontractility under high catecholamine surges [60,62]. Hyperdynamic myocardial tissue over the LV base as the fundamental tissue aspect is included into the diagnostic criteria in acute stress cardiomyopathy [22,23]. Interestingly, basal predominance and impairment in apical tissue metabolism on positron emission tomography (PET) are reversible and completely resolved after acute episode [63]. Viewed through this integrated model, Takotsubo syndrome is not a purely reactive phenomenon, but a predictable expression of myocardium rendered fragile by chronic maladaptive remodeling. This framework explains cases occurring in the absence of overt emotional stress, accounts for disease recurrence in susceptible individuals, and emphasizes the importance of identifying the chronic substrate using modalities such as stress echocardiography. Recognizing Takotsubo syndrome as the acute endpoint of long-standing myocardial susceptibility carries significant implications for diagnosis, risk stratification, and long-term management, shifting clinical focus toward early detection and substrate-directed preventive strategies.

## 6. Tissue Energetics and Adaptation in AS

**Figure 4:**
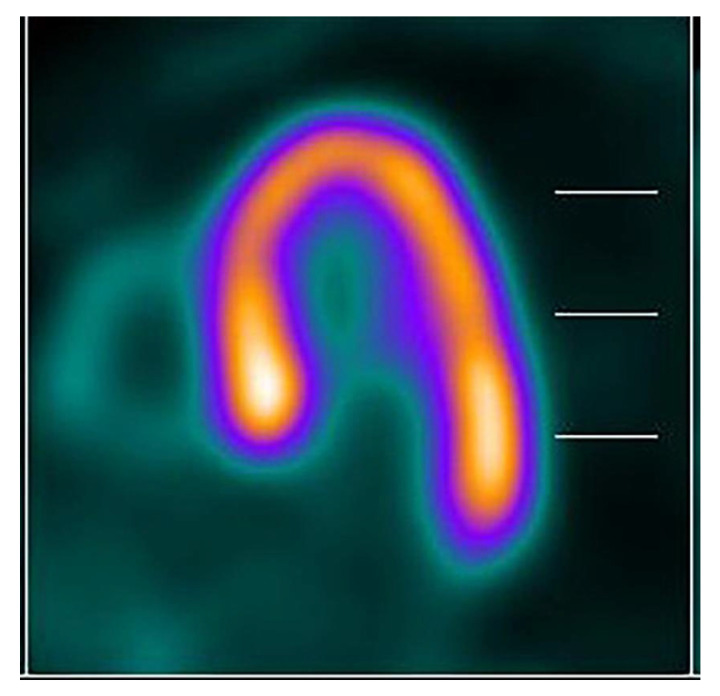
Long horizontal axis of PET image shows an increased metabolism in septal base compared to the other myocardial segments in a hypertensive patient.

**Figure 5:**
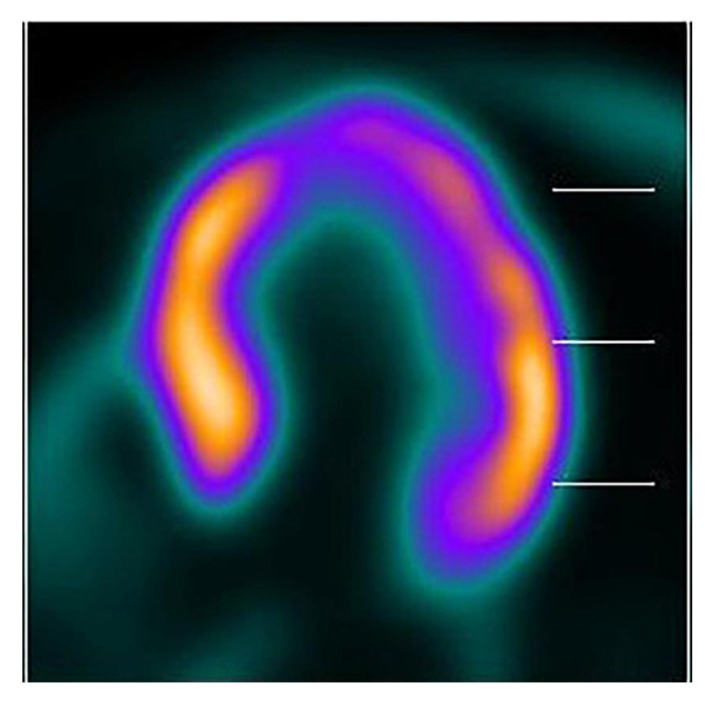
Long horizontal axis PET image of a patient with severe AS and SHM. The scan reveals remarkable SHM with hypermetabolic activity at the septal base, which decreases towards the mid-septal segment, contrasting with severely suppressed metabolism at the apex. This pattern represents an advanced phase of remodeling.

**Figure 6:**
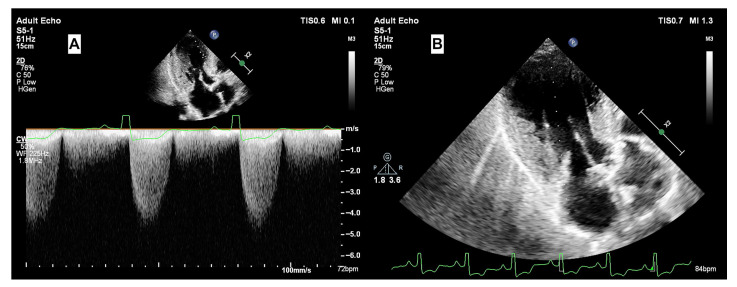
Hemodynamic and structural assessment of the same patient. (**A**) Increased transvalvular aortic gradient measurements. (**B**) Visualization of the predominant septal base accompanied by a relatively larger apical cavity during diastole, consistent with the remodeling patterns observed in severe AS.

**Figure 7:**
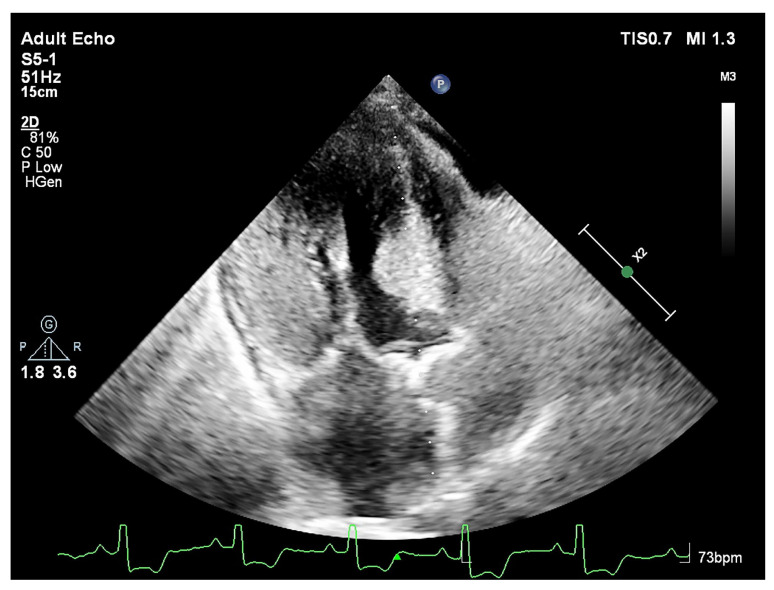
Systolic phase imaging of the same patient. The remarkably thickened LV base results in the protrusion of the septal base (SHM) into the LV outflow tract. The image provides a striking view of the demolished apical tissue at systole, highlighting the distinct structural consequences of this advanced remodeling phase.

Chronic obstruction in AS drives progressive LV hypertrophy. While this process is initially compensatory, sustained pressure overload ultimately promotes maladaptive remodeling and the transition to heart failure [61]. Advanced metabolic imaging has provided critical insight into this progression. PET imaging consistently reveals a hypermetabolic septal base in SHM. This metabolic pattern persists regardless of the etiology, whether triggered by functional stress in hypertension (Figure 4) or chronic mechanical-based stimulus in AS patients (Figure 5). In severe AS, this remodeling manifests as a hyperdynamic base (Figure 6) contrasted with severe apical hypokinesis and reduced metabolic uptake (Figure 7). This pattern highly mirrors the apical dysfunction seen in acute stress cardiomyopathy.

These imaging findings support a unified mechanistic cascade linking hemodynamic burden to clinical deterioration. Chronic pressure overload imposed by the stenotic valve alters regional left ventricular wall stress distribution, with preferential mechanical loading of the basal septum due to ventricular geometry and direct exposure to the high-velocity outflow [64]. This regional stress concentration markedly increases local myocardial work and oxygen demand. At the same time, elevated intracavitary pressures and reduced coronary perfusion reserve compromise subendocardial blood flow, creating a microvascular supply–demand mismatch [65]. In the early adaptive phase, this mismatch is compensated by upregulation of glucose utilization, producing the hypermetabolic basal phenotype observed on FDG PET. With continued pressure overload, progressive microvascular dysfunction, mitochondrial inefficiency, and impaired substrate flexibility drive a transition from adaptive metabolic augmentation toward metabolic exhaustion. This metabolic deterioration activates profibrotic signaling, leading to increasing interstitial fibrosis, loss of regional compliance, and progressive impairment of contractile reserve [66,67]. Ultimately, the combined effects of metabolic failure and fibrotic remodeling compromise global ventricular performance, manifesting clinically as diastolic dysfunction, reduced exercise tolerance, and heart failure symptoms.

We have recently suggested that this segmental discrepancy could be extremely important and the main reason of heart failure development [68]. Historically, untreated severe AS complicated by heart failure carried a dismal prognosis. However, survival rates are now understood to vary significantly based on the specific stage of cardiac damage and the timing of intervention [69,70]. These findings support the notion that SHM represents a predominant and hypermetabolic septal base for stress adaptation, while the mid-apical part has a remarkably defective metabolism.

The regional pattern of PET glucose uptake, characterized by increased metabolic activity in the basal septum with progressive decline through the mid-septum and severe suppression at the apex, observed in patients with severe aortic stenosis and coexisting hypertension appears to reflect a structured progression of pressure overload remodeling rather than a simple imaging finding [64]. This spatial metabolic gradient likely represents the transition from early compensatory metabolic adaptation to advanced metabolic failure accompanied by fibrotic decompensation [71]. This concept is further supported by longitudinal observations in hypertensive heart disease, where myocardial Fluorine-18 fluorodeoxyglucose (FDG) uptake initially increases during the early adaptive phase of pressure overload but progressively declines as hypertension advances to left ventricular hypertrophy and diastolic dysfunction [72]. Importantly, noninvasive FDG PET imaging has been shown to predict this transition, providing an opportunity for early identification of maladaptive remodeling and the potential to halt disease progression through aggressive therapeutic intervention. This metabolic decline appears to be driven in part by progressive myocardial fibrosis accompanying the transition from compensatory hypertrophy to structural decompensation [71,73]. Notably, this pattern is more pronounced in patients with combined aortic stenosis and hypertension than in those with hypertension alone, suggesting a synergistic effect of dual pressure overload on myocardial metabolic deterioration [74]. Integration of serial PET imaging with longitudinal clinical outcomes and histopathologic correlation in future studies may establish metabolic phenotyping as a robust risk stratification strategy in aortic stenosis, enabling a shift from reliance on isolated hemodynamic thresholds toward individualized, metabolism guided timing of intervention aimed at maximizing myocardial preservation and functional recovery.

## 7. Consistency Between Clinical and Animal Validation Data

Animal validation studies demonstrated that distinct stress stimuli, whether physiologic or pathologic, produce convergent myocardial responses. These findings suggest that the observed clinical phenomenon reflects the integrated effect of multiple stress inputs, providing a mechanistic basis for the concept of SHM. Importantly, SHM appears to represent a broader clinical spectrum that is not limited to hypertension as a functional consequence of increased afterload but also encompasses conditions for instance acute stress cardiomyopathy driven by emotional stress and aortic stenosis driven by mechanical overload. SHM is a validated marker of the adaptive phase of left ventricular remodeling and is consistently associated with increased fluid and tissue dynamics under stress across both human and animal studies [17,18,19,20,27,28,30,54].

Both experimental and human studies demonstrate a marked apical to basal neurohumoral gradient within the left ventricle. The basal myocardium contains significantly higher concentrations of norepinephrine and exhibits denser sympathetic innervation compared with the apical region, whereas the apex is characterized by the highest density of β-adrenergic receptors but relatively sparse sympathetic nerve supply [75,76]. This intrinsic spatial heterogeneity of adrenergic signaling establishes a structural and functional substrate for regionally distinct myocardial responses to stress. Pharmacologic modulation of this system further supports its clinical relevance, as angiotensin receptor blockade and beta-blockers has been shown to significantly reduce circulating epinephrine and norepinephrine levels [77]. Collectively, these observations provide a mechanistic framework through which elevated catecholamine exposure may drive region-specific stress-related myocardial remodeling in both acute and chronic cardiac conditions.

A consistent finding was reported that cardiac metabolism was reasonably preserved at the LV hypertrophy stage before heart failure in Dahl salt-sensitive rats [39]. Predominant septal base in SHM as the adaptive tissue to a severely resistant valve may need more aggressive intervention [78].

As a conclusion, hemodynamic stress, which is independent from the stressor, results in SHM, which is the early imaging biomarker of LV remodeling and possibly represents an adaptive phase of LV remodeling. Predominant basal tissue and energetics due to hypermetabolism in SHM could be more striking and related to long-term hemodynamic overload in AS.

## 8. Limitations and Future Directions

Although this review study presents a cohesive mechanistic and imaging-based framework for understanding the Stress Septal Sign and its associated metabolic phenotype, several limitations should be acknowledged. Addressing these limitations is essential for strengthening the clinical applicability of the proposed concepts.

The majority of the evidence supporting the Stress Septal Sign and its metabolic signature is derived from cross-sectional imaging observations. While these data consistently demonstrate strong associations between myocardial structure, function, and metabolism, cross-sectional analyses inherently limit causal inference regarding disease progression. Although existing findings support a staged transition from early adaptive remodeling toward metabolic decompensation, more prospective longitudinal investigations are required to formally establish temporal relationships and to define clinically meaningful thresholds for disease progression.

The frequent coexistence of systemic hypertension in patients with aortic stenosis represents a significant confounding factor. Hypertension independently promotes myocardial hypertrophy, fibrosis, and metabolic alterations, thereby complicating the attribution of observed remodeling patterns solely to valvular obstruction. Although the present data suggest that metabolic deterioration is more pronounced when aortic stenosis and hypertension coexist, future studies incorporating rigorously characterized patient populations and comprehensive multivariable analyses will be necessary to isolate the relative contributions of mechanical valvular load and systemic pressure overload.

While the current framework highlights the potential of metabolic imaging to refine risk stratification beyond conventional hemodynamic measures, prospective outcome-based trials evaluating metabolism-guided therapeutic strategies in aortic stenosis are still lacking. Large-scale longitudinal studies combining serial metabolic imaging with clinical endpoints, ventricular remodeling metrics, and survival outcomes will be required before such an approach can be incorporated into routine clinical decision-making.

## Data Availability

No new data were created or analyzed in this study.

## Abbreviations

The following abbreviations are used in this manuscript:

LV	Left Ventricle
BSH	Basal Septal Hypertrophy
LVH	LV Hypertrophy
AS	Aortic Stenosis
Triple S	Stress Septal Sign
PET	Positron Emission Tomography
SHM	Stressed Heart Morphology
